# Influence of Anhydrite on the Properties and Microstructure of Aluminophosphate Cement

**DOI:** 10.3390/ma15197005

**Published:** 2022-10-09

**Authors:** Zhu Ding, Jiongchao Chen, Shuqing Zheng, Yue Hu, Yuan Fang

**Affiliations:** Key Laboratory of Durability of Coastal Civil Engineering of Guangdong Province, School of Civil Engineering, Shenzhen University, Shenzhen 518060, China

**Keywords:** hydraulic cementitious material, aluminophosphate cement, anhydrite, compressive strength, pore analysis

## Abstract

Aluminophosphate cement (APC) is a new type of hydraulic cementitious material with many potential functions. Microscopic analysis techniques were used to study the effect of anhydrite on the performance and hydration process of APC under standard curing conditions. The results show that adding an appropriate amount of anhydrite promotes the hydration of APC. The highest compressive strength is reached at an anhydrite content of 15 wt.%. As the anhydrite content increases, the APC’s compressive strength decreases. The microscopic analysis of the hydration product morphology shows that the incorporation of anhydrite produces ettringite and other hydration products, improving the microstructure of the cement paste. The mercury intrusion porosimetry results show that the total porosity of the hardened APC paste decreases, and the microstructure becomes denser with an increase in the curing age, resulting in an increase in the compressive strength over time.

## 1. Introduction

Ordinary Portland cement (OPC) is the most common building material in today’s construction engineering and a crucial component of infrastructure projects and the developing economy. Since the civil construction environment has become more complex, cement performance requires improvement [1]. OPC clinker consists of C_3_S, C_2_S, C_3_A, and C_4_AF. Calcium silicate hydrate (C-S-H), calcium hydrate (CH), and calcium sulfoaluminate hydrate (ettringite, AFt) are present in OPC after hydration and hardening. A cement paste with this composition is prone to shrinkage, creep, and chemical erosion, especially in coastal and other corrosive environments. Therefore, it is necessary to develop improved new cement materials for structures in some special environments [2]. Cementing materials, such as geopolymer cement [3,4], aluminate cement [5], sulfoaluminate cement [6,7], and phosphate cement [8,9], can replace OPC to build structure in, for example, marine environments. Aluminate cements (CAs) have the characteristics of fast hardening, early strength and good high temperature resistance, but their long-term strength will produce shrinkage [10,11]. Sulfoaluminate cement (SAC) has the characteristics of fast hardening and early strength, and can be used as a micro expansion cement. However, when the temperature or alkalinity changes, the carbonation resistance of hydration products deteriorates, and the durability and chemical stability are limited [12]. Recently, the invention of aluminophosphate cement (APC) with high strength both early and later, as well as excellent durability, has further improved people’s understanding of cementitious materials, and promoted the progress of cement preparation technology.

APC was developed based on the CaO-Al_2_O_3_-P_2_O_5_ ternary system [13,14]. APC clinker consists of calcium aluminate (CA), tricalcium phosphate (C_3_P), calcium aluminophosphate (CAP) mineral phases, and a small amount of glass phase. It has the advantages of high early and late strength and higher chemical stability and durability than OPC and sulfoaluminate cement. Research on its anti-permeability mechanisms has shown that the hardened APC paste has a different pore system than OPC [15,16].

Both OPC and APC contain calcium aluminate; the former contains C_3_A, and the latter contains CA. If no retarder is used when water is mixed with OPC, it sets too quickly. Generally, OPC requires gypsum as a retarder to react with C_3_A to form ettringite during the hydration and hardening of OPC. However, in early studies on APC, the retarder was not gypsum [17]. When APC was added as an admixture to OPC, the APC accelerated the hydration rate of OPC [18], enhancing its strength. The mechanical properties and frost resistance of Portland cement can be improved by adding APC clinker to Portland cement after modification [19]. When APC and OPC are mixed, the aluminates content is increased in the hydration system, increasing the content of hydrated aluminate products and improving the microstructure and permeation resistance [20]. APC has excellent properties, including frost resistance, high-temperature resistance, and good reinforcement performance [21,22,23,24].

The main hydration products in the hydrated APC paste are calcium aluminophosphate hydrate (C-A-P-H) and calcium aluminate hydrate (C-A-H) gels. Small quantities of crystal products, hydrated calcium phosphate, and hydrated calcium aluminate (C_2_AH_8_) are also present [25]. These hydration products of APC react with each other and fill the pores of the hardening paste, decreasing the pore size, improving the density, and reducing porosity. This microstructure facilitates the bonding with chloride ions [26]. Therefore, APC may be a promising cementitious material and an important addition to Portland cement.

Since APC is a relatively new cement type, its performance and microstructure have not been investigated in-depth, as regards APC clinker preparation, performance, composition, hydration, hardening, mechanical property development, and durability. This paper focuses on the relationship between the performance and composition of APC, especially the effect of gypsum on APC’s compressive strength development. It is known that gypsum has a retarding effect on Portland cement. However, the effect of gypsum on the hydration of APC has not been analyzed to date. The objective of this paper is to evaluate the influence of anhydrite on the setting, compressive strength development, and hydration of APC. The results of this study provide new insights into the hydration mechanism and microstructure of APC.

## 2. Experimental

### 2.1. Raw Materials and Mix Ratio

The APC used in this study was synthesized in the laboratory using the sol-gel method and high-temperature calcination. The weight ratio of CA to C_3_P to CAP was 20:30:50. The chemical reagents used in the experiment were chemically pure calcium nitrate [Ca(NO_3_)_2_•4H_2_O, 99%], phosphoric acid (H_3_PO_4_, 85%), and aluminum nitrate [Al(NO_3_)_2_•9H_2_O, 99%]. Deionized water was purchased from Shenzhen Bule Industrial Co., Ltd. (Shenzhen, China) Anhydrite (CaSO4, 97%) was purchased from Shanghai Maclean Company (Shanghai, China). Four binder samples were prepared, one was the APC clinker itself, the other three samples were APC clinker blended with different dosage of anhydrite. The mix ratio is listed in Table 1.

### 2.2. Test Methods

#### 2.2.1. APC Clinker Preparation Process

**Figure 1:**
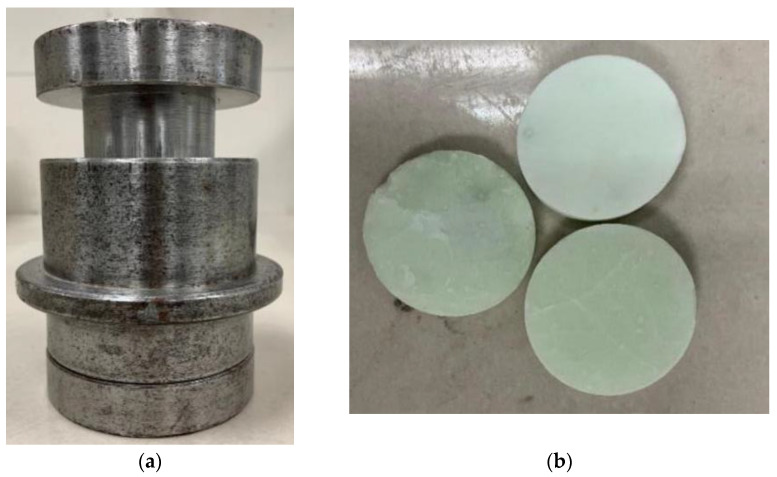
Mold and clinker sample; (**a**) Mold; (**b**) Tablets.

The preparation method of APC clinker is as follows. A sol was prepared according to the APC clinker mix ratio. The beaker containing the reagent material was put into the thermostatic water bath, and the temperature of the thermostatic water bath was maintained at about 90 °C. Then the cantilever stirrer was fixed with the iron bracket and the cross clip, and the beaker was stirred at a constant speed, maintained at 215 r/min. After most of the water in the beaker evaporates, the gel becomes sticky. The sticky gel was taken out of the beaker and placed in an iron tray. It was then placed in a high temperature blast oven (model DHG-9030A), with the temperature set at 300 °C for 24 h until the gel became fluffy and dry. The dried gel was removed and placed in a high-temperature resistance furnace (model SSG-12-16C, Shanghai Yifeng Electric Furnace Co., Ltd., Shanghai, China) for pre-burning at 900 °C for two hours. It was removed and left to cool to room temperature, followed by placing it in a high-speed crusher (model RHP-600A) and grinding it into a powder with a size of 200 mesh. A mold and a press machine (YZH-300.10 type, Luda Machinery Instrument, Shaoxing, China) were used to press the powder into a round cake with a diameter of 5 cm and a thickness of 1–2 cm. The cakes were placed into the high-temperature resistance furnace at 1590 °C for two hours. Subsequently, they were removed and left to cool to room temperature in the air. The mold and APC clinker samples are shown in Figure 1. The clinker was ground with a high-speed grinder, and the ground powder was passed through a 325-mesh standard sieve. Then, the APC clinker powder was sealed in a plastic bag prior to the analysis.

#### 2.2.2. APC Setting Time and Compressive Strength Tests

**Figure 2:**
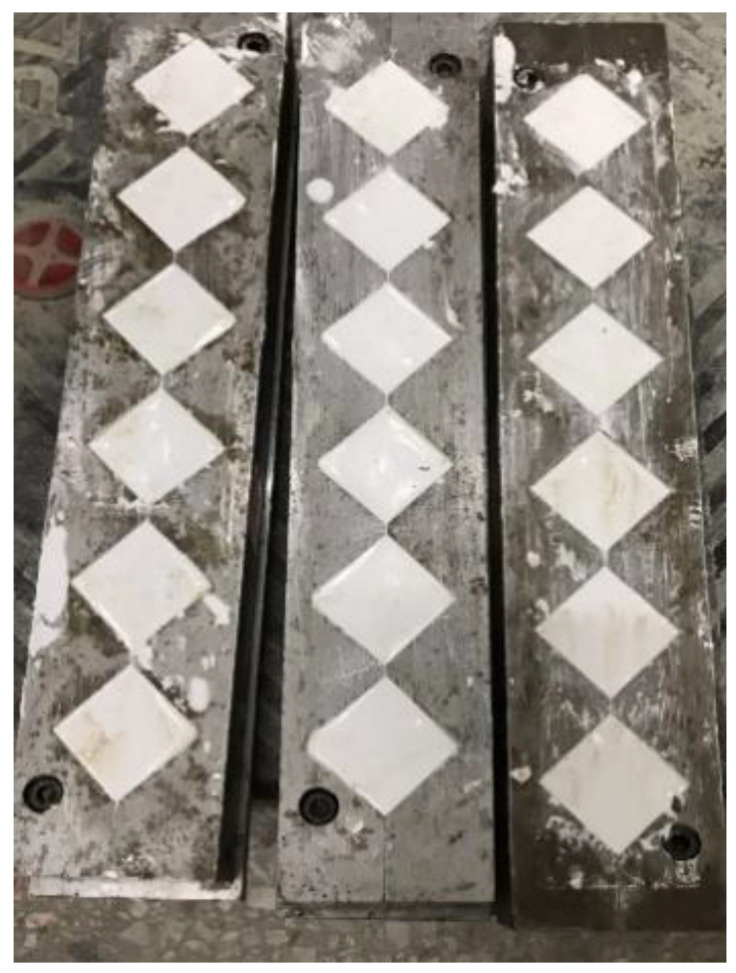
Test samples of the APC and anhydrite.

The setting time of the APC was tested using the GB/T1346-2001 standard. We used a standard Vicat meter to test the initial and final setting times of the APC paste. The initial setting time was tested every 5 min. The final setting time was tested by placing the specimen in a moisture curing box and examining it every 15 min. The compressive strength of the hardened APC paste was measured using the GB/T17671-1999 standard. The APC paste sample was a cube with a dimension of 30 mm × 30 mm × 30 mm. The water-binder ratio of all pastes was 0.3 (Figure 2). The specimens were cured under standard conditions (20 °C ± 1 °C, RH ≥ 90%). The compressive strength of the APC specimens of different ages was evaluated using a constant-load cement compressive testing machine. Each group consisted of three samples, and the average value represented the specimen’s strength.

#### 2.2.3. Microstructure Analysis

X-ray powder diffraction (XRD) (D8 Advance, Bruker, Billerica, WA, USA), scanning electron microscopy (SEM) (FEI Corporation, Quanta TM 250 FEG3040702, Hillsboro, OR, USA), and thermogravimetry-differential thermal analysis (TG-DTA) (STA 409PC) were used to analyze the microstructure of the hardened APC paste.

## 3. Results and Discussion

### 3.1. Setting Time and Compressive Strength of APC

**Figure 3:**
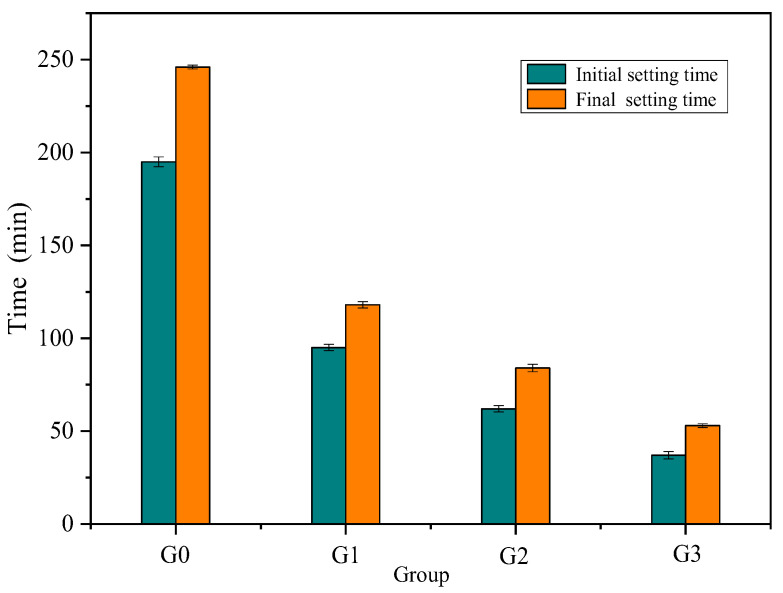
Influence of anhydrite content on the setting time of APC.

The results of the setting time are shown in Figure 3. As the anhydrite content increased, the initial and final setting times of the APC decreased because the anhydrite promoted the hydration of the APC. The XRD results (see Section 3.2) indicated that ettringite (3CaO•Al_2_O_3_•3CaSO_4_•32H_2_O, or AFt for short) had formed due to the reaction of APC and anhydrite [27], accelerating the formation of a skeleton in the cement paste because of acicular crystals. Portland cement sets quickly if no gypsum is added due to the rapid hydration of C_3_A. The addition of gypsum to Portland cements results in the formation of ettringite due to the reaction of gypsum and C_3_A [28], retarding the hydration reaction. The effect of gypsum on different cements is a topic worth further investigation.

**Figure 4:**
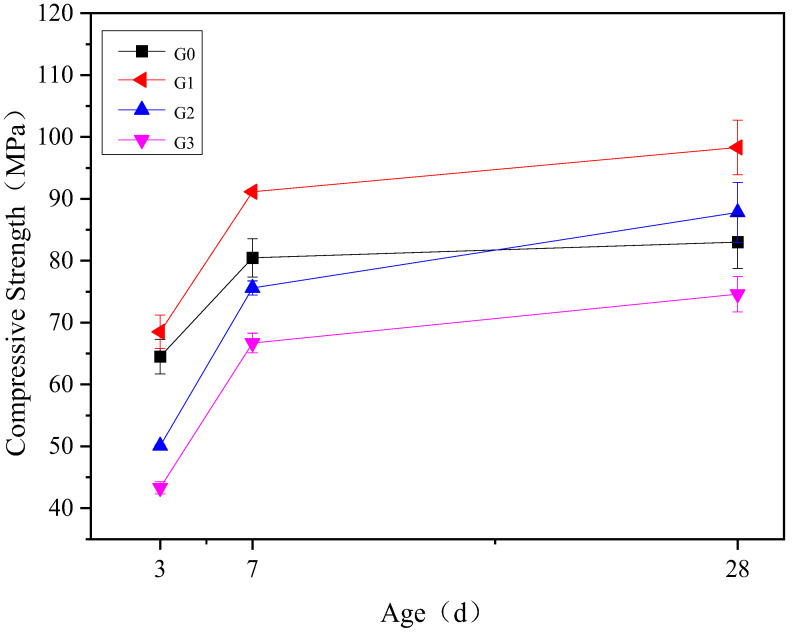
Compressive strength of APC pastes with different anhydrite contents.

The water-binder ratio of the APC used in the compressive strength test was 0.3. The APC samples in different groups were tested at the curing ages of 3, 7, and 28 d. The results are shown in Figure 4. The addition of gypsum changes the hydration reaction rate and the compressive strength development [29]. The compressive strength at 3, 7, and 28 d is the highest at 15% anhydrite. The compressive strength of the G1 group is nearly 70 MPa at 3 d and 98.3 MPa at 28 d. It increased rapidly in the early stage, but the growth rate decreased in the later stage of hydration. The compressive strength of the G0 group increased rapidly in the early stage, reaching 80.6 MPa after 7 d. Subsequently, it increased slowly and was only 3.6% higher at 28 d than at 7 d. The anhydrite content of the G2 group was 20%. The compressive strength was lower than that of the G0 group at 3 d and 7 d but almost the same as 28 d. Although the compressive strength of the G2 group was relatively low in the early stage, its growth rate was higher than that of the G0 group between 3 d and 7 d, and there was a negligible difference in the 7 d compressive strength between the G2 group and the G0 group. The compressive strength at 28 d and its growth rate from 7 d to 28 d were slightly higher for the G2 group compared to the G0 group. The anhydrite content of the G3 group was 25%. The compressive strength was lower at each age than that of the other groups under standard curing conditions.

The compressive strength development results indicate that an appropriate dosage of anhydrite promoted the hydration of APC. The compressive strength of the G1 group with an anhydrite content of 15% was the highest at all curing ages. At age 28 d, its compressive strength was nearly 20% higher than the sample without any anhydrite.

### 3.2. XRD Results of Hardened APC Paste

**Figure 5:**
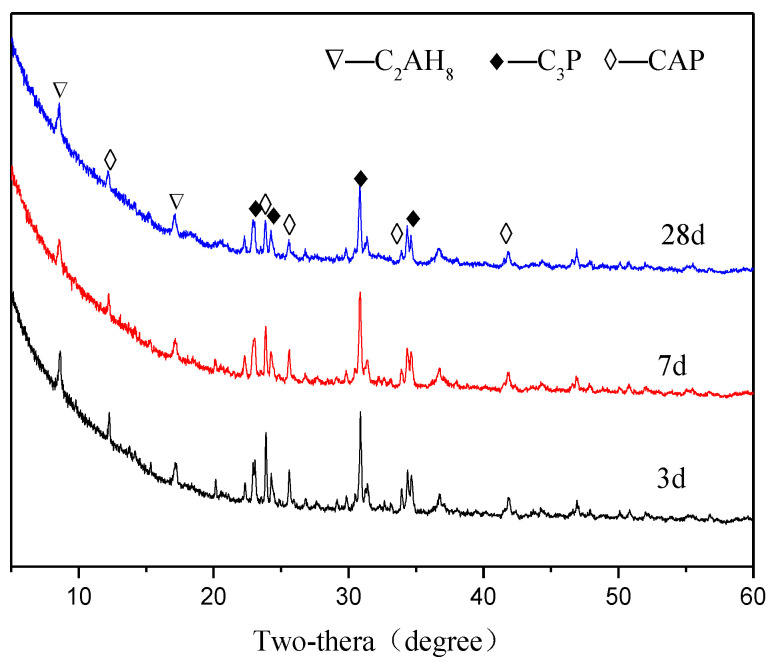
XRD patterns of APC paste for G0 group at different ages.

**Figure 6:**
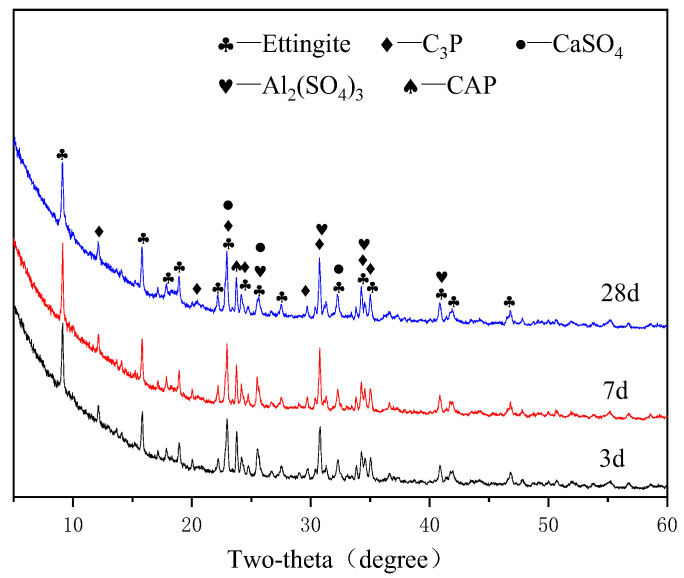
XRD patterns of APC paste for G1 group at different ages.

**Figure 7:**
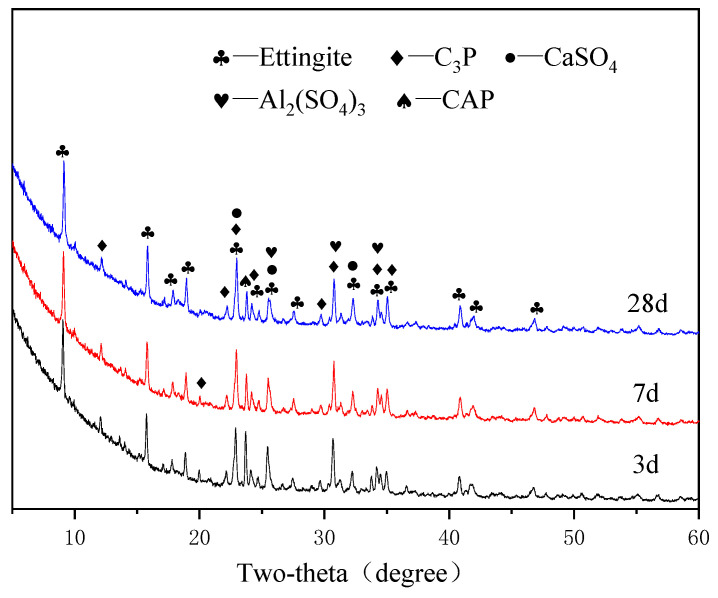
XRD patterns of APC paste for G2 group at different ages.

**Figure 8:**
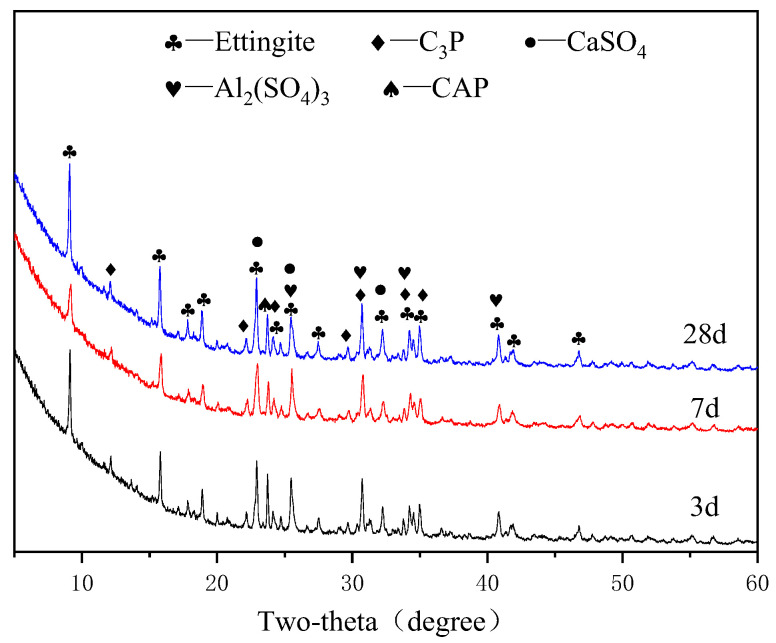
XRD patterns of APC paste for G3 group at different ages.

Figure 5, Figure 6, Figure 7 and Figure 8 show the XRD patterns of the APC hydration products for different ages.

Figure 5 shows the XRD patterns of the APC hydration products for the G0 group at different ages. The crystal phase of the hardened APC paste includes calcium aluminate hydrate (C_2_AH_8_) (PDF#45-0564), C_3_P (PDF#09-0348), and CAP. The main diffraction peak of CAP is at 2θ = 23.643°. The intensities of the diffraction peaks of C_2_AH_8_ increase slowly with the curing age, indicating the formation of hydration products. The intensities of the diffraction peaks of C_3_P and CAP decrease with the curing age, demonstrating that C_3_P and CAP are continuously hydrated. Amorphous phases also formed in this system, such as C-A-P-H and A-P-H gels, but their diffraction peaks are not visible in the XRD spectra.

Figure 6, Figure 7 and Figure 8 show the XRD patterns of the APC specimens in the G1, G2, and G3 groups at different ages. It can be concluded that the hydration products of APC are the same, although they have different anhydrite contents. The main crystal products of the three groups of samples after the hydration reaction of APC and anhydrite are ettringite (PDF#41-1451), Al_2_(SO_4_)_3_ (PDF#18-0060), and other products. The chemical reactions for the ettringite formation is shown in Equations (1)–(3). When APC clinker and gypsum are mixed with water, they quickly dissolve Ca^2+^, OH^−^, SO_4_^2−^, and AlO_2_^−^ because APC contains CA. Then, these ions combine to form [Ca_6_(Al(OH)_6_)_2_•24H_2_O]^6+^, which combines with water and SO_4_^2−^ to form ettringite.

AlO_2_^−^ + 2H_2_O → Al(OH)_4_^−^ (1)

2Al(OH)_4_^−^ + 4OH^−^ + 6Ca^2+^ + 24H_2_O→ (Ca_6_(Al(OH)_6_)_2_•24H_2_O)^6+^ (2)

[Ca_6_(Al(OH)_6_)_2_•24H_2_O]^6+^ + 3SO_4_^2^^−^ + 2H_2_O→ 3CaO•Al_2_O_3_•3CaSO_4_•32H_2_O (3)

The hydration process of C_3_A substantially affects the setting of Portland cement [30], and the significant viscoelastic development of Portland cement after the addition of gypsum is attributed to the generation of ettringite. The XRD patterns show that the ettringite has the highest diffraction peaks, indicating the formation of large amounts of ettringite after the addition of anhydrite because of the high contents of CaSO_4_ and CA. The formation of ettringite changes the hydration route of APC and accelerates its setting and hardening. The peaks of ettringite increase with the curing age, demonstrating that ettringite is being formed after the hardening of the APC paste. In addition, the XRD pattern shows the presence of CaSO_4_ (PDF#03-0163), C_3_P, and CAP. The intensity of the main diffraction peak of CaSO_4_ decreases with an increase in the curing age, indicating that CaSO_4_ is involved in the hydration reaction of APC. Therefore, the intensity of the diffraction peak of CaSO_4_ is lower in the later stage of the hydration reaction. The presence of CaSO_4_ is due to the anhydrous gypsum that was added and incomplete reaction.

### 3.3. Micromorphology of the Hardened Paste

**Figure 9:**
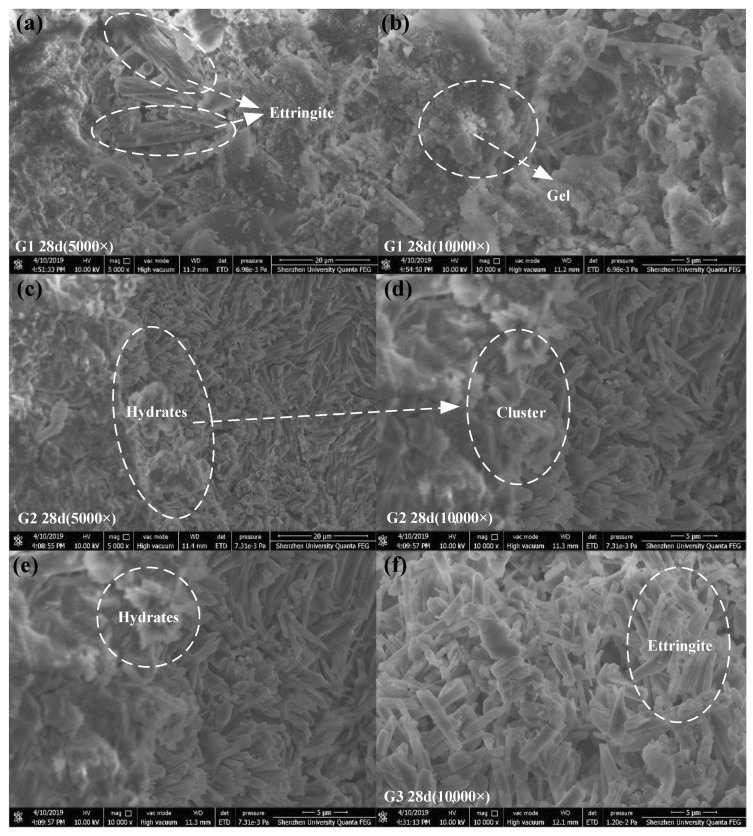
SEM images of the microscopic morphology of the G1, G2, and G3 samples at 28 d. (**a**,**b**) SEM image of G1 sample at 28 d; (**c**,**d**) SEM image of G2 sample at 28 d; (**e**,**f**) SEM image of G3 sample at 28 d.

The hardened APC paste samples of different ages were analyzed by SEM to observe the microstructure and morphology characteristics. Figure 9a,b show the SEM images of the microscopic morphologies of the G1 group at different magnifications at 28 d. As the curing age increases, the hydration products become denser. At 28 d, there are few rod-like hydration products on the surface but many gel products surrounding the rod-like hydration products, resulting in a continuous structure. Some needle-like and columnar hydration products are observed on the sample surface; these are ettringite. Similar to the results of the mechanical property analysis, the G1 group has the highest 28-d compressive strength due to its microstructure.

Figure 9c,d show the SEM images of the G2 group at 28 d at different magnifications. There are few pores and cracks on the surface, and the hydration gel is connected to the crystals, forming a dense and continuous structure. At 10,000× magnification, the thickness of the needles has changed slightly. Therefore, as the curing age increased, the morphology and crystal size of the hydration products in the G2 group only changed slightly.

Figure 9e,f show different magnifications of the SEM images of the G3 group at 28 d. The distribution, size, and length of the columnar hydration products differ for different curing ages. A substantial amount of gel has formed in the APC paste and has become part of the columnar hydration products, resulting in a dense and continuous structure. These columnar hydration products, which are ettringite, are randomly distributed in the paste. However, the length of the ettringite columns is shorter than that of the other samples; therefore, the encapsulated flocculent gel has worse properties than that of the other groups. This result is the likely reason for the lower strength of the G3 sample.

The density of the cement paste affects its mechanical properties [31]. The compressive strength of G3 is the lowest, which is consistent with the microstructure of hydration products.

Compared with samples G2 and G3, in sample G1, the ettringite crystal size is larger and more gel products are formed, in which the crystals as the framework are interwoven with gels into a more continuous and solid microstructure.

### 3.4. Pore Structure of the Hardened Paste

The hydration products and hydration mechanism of cement concrete provide a specific microstructure and pore characteristics. The pore structure characteristics significantly affect the permeability, air tightness, frost resistance, thermal conductivity, strength, stiffness, and toughness of cement concrete materials [32]. Some researchers used silicate-sulfoaluminate cement to prepare thermal insulation foamed concrete. The smaller the pore size, the more uniform were the distribution, the higher the compressive strength, and the lower the thermal conductivity [33]. It has been demonstrated [34] that the hydration process of APC is a dynamic equilibrium process driven by the synergistic effect of chemical reactions, such as the dissolution of gelled minerals and the crystallization of hydration products. This process can be assessed by analyzing the pore structure. The unique properties and hydration mechanism of APC provided it with high early strength.

Since the compressive strength of the APC mixed with 15% anhydrite was the highest, the G0 and G1 groups were selected for the mercury injection test to conduct pore analysis. The effect of hydration time on the pore size of APC and APC-anhydrous calcium sulfate pastes was investigated. In Portland cement-based materials, the pore size is typically divided into the following types: more harmful pores are greater than 200 nm, harmful pores are between 50 nm and 200 nm, less harmful pores are between 20 nm and 50 nm, and harmless pores are less than 20 nm [35].

**Figure 10:**
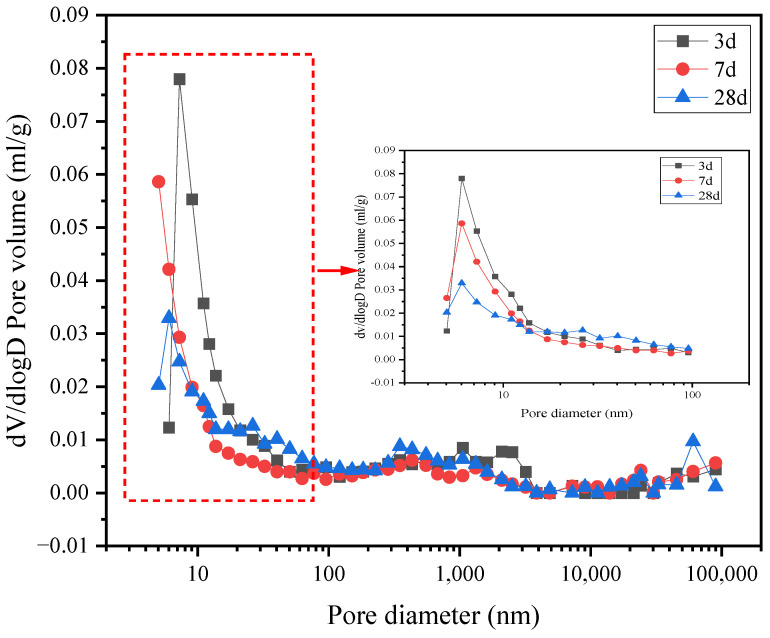
Differential curve of the pore size distribution of the G0 paste.

**Figure 11:**
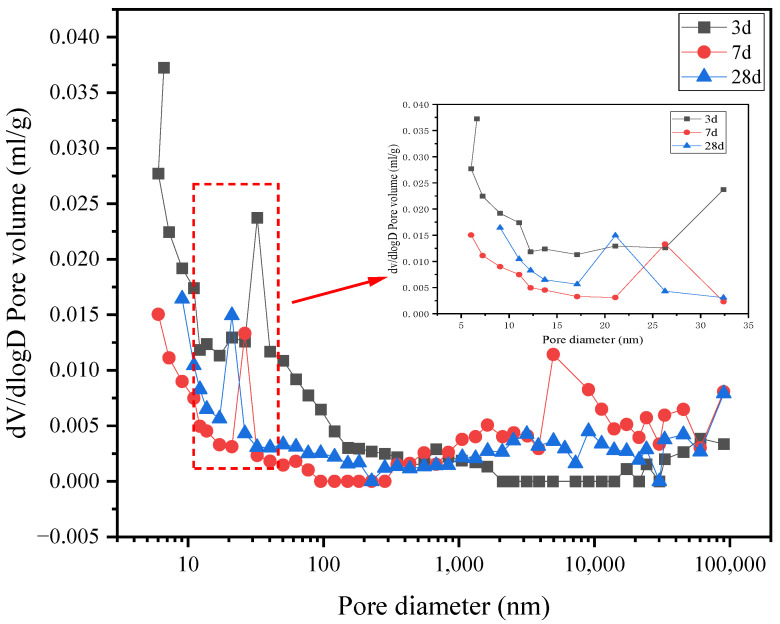
Differential curve of the pore size distribution of the G1 paste.

Figure 10 and Figure 11 show the differential curves of the pore size distribution of groups G0 and G1. The largest proportion of the pore sizes of group G0 is below 10 nm, indicating harmless pores. Although G0 has pores > 200 nm, the proportion is relatively small. The largest proportion of the pore size of the G1 group is between 21 nm and 33 nm at different curing ages, suggesting less harmful pores. As the curing age increases, the maximum pore size decreases, and the paste becomes denser, indicating progress in the hydration of the G1 group. The differential curve of the G1 sample shows that the amount of mercury in the pores is small, and the injected mercury mainly appears in 3 d. These results combined with the compressive strength and XRD results demonstrate that the formation of ettringite after mixing the APC with the anhydrite resulted in a dense microstructure.

**Figure 12:**
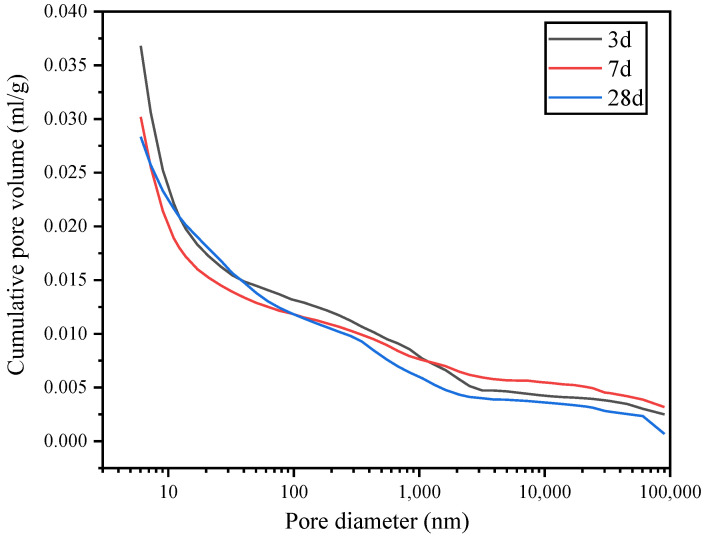
Cumulative pore volume of the G0 sample.

**Figure 13:**
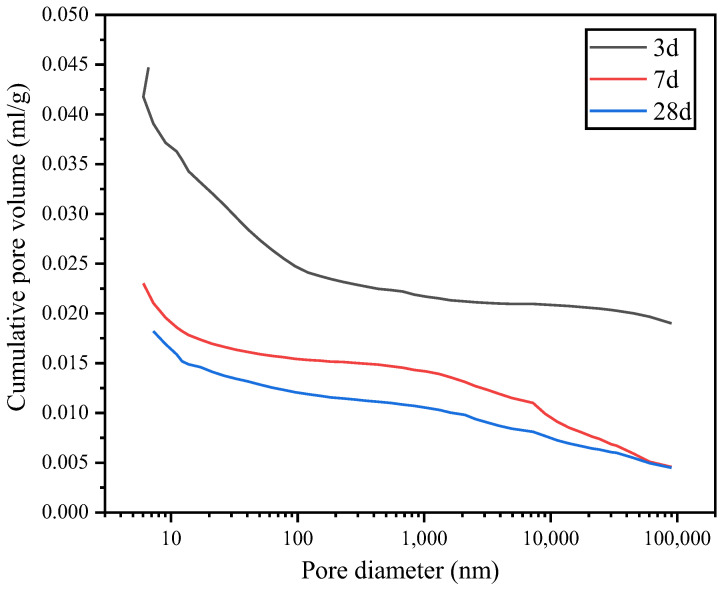
Cumulative pore volume of the G1 sample.

Figure 12 and Figure 13 show the cumulative pore volume of the hydrated paste in the G0 and G1 samples, respectively. The pore size range was 6–90 nm. Figure 12 indicates that the cumulative mercury content of the G0 sample is 0.0368 mL/g at 3 d, 0.0302 mL/g at 7 d, and 0.0283 mL/g at 28 d. As the curing age increases, the cumulative mercury content in the pores of the G0 paste decreases, indicating that the pore size decreases, and the structure becomes more compact. The inflection point of the curve indicates the critical pore size, which is 21.094 nm, 17.105 nm, and 17.096 nm at 3, 7, and 28 d, respectively.

As the curing age increases, the critical pore size of the G0 paste decreases, and the hydrated paste becomes denser. Therefore, the permeability and durability of the hydrated paste improves. Figure 13 shows that the Hg content in the G1 paste is 0.0472 mL/g at 3 d, 0.0210 mL/g at 7 d, and 0.0182 mL/g at 28 d. The Hg content in group G1 is significantly higher at 3 d than at 7 d and 28 d, indicating the largest number of pores in the early hydration stage in group G1. With the increase in the curing age, the hydration products in the paste fill the internal pores. Therefore, the volume of pores in the paste decreases significantly from 3 d to 7 d and then further decreases as the interior of the hydrated paste becomes optimized. Therefore, the compressive strength of the G1 paste is higher at 7 d and 28 d than at 3 d.

In this study, the results of MIP porosity show the changing pore structure of the hardened APC cement paste with time. For example, the pattern of the pore size distribution and total porosity changes with time from 3 d to 28 d. After blending with anhydrite, the total porosity reduces with time, and the changes correspond to the strength development.

## 4. Conclusions

The influence of different anhydrite contents (0, 15%, 20%, and 25%) on the compressive strength of APC under standard curing conditions was investigated. The results provide some theoretical support that anhydrite can improve the strength properties of APC. The conclusions are as follows.

1. As the anhydrite dosage increased from 15% to 20% and 25%, the setting time of the APC decreased. The reason is that the formation of ettringite through the reaction of APC and anhydrite accelerated the formation of a dense microstructure in the cement paste. Adding anhydrite to APC changes the hydration reaction mechanism.
2. The optimum gypsum dosage to obtain the highest compressive strength was 15% at 28 d under normal curing conditions. In this case, the ettringite crystal size is larger and more gel is formed, which provides a more reasonable microstructure. When the dosage of gypsum exceeded 15%, the strength decreases.
3. The main hydration products of APC without anhydrite were C_2_AH_8_ and gel products. Ettringite formed after the addition of gypsum, with a framework bonded by gels into more continuous and solid microstructure. The results of the mercury intrusion porosimetry test showed that as the porosity of the APC paste decreased, especially when blended with suitable anhydrite, the microstructure became denser as the curing age increased. This trend corresponds to increasing strength.

## Figures and Tables

**Table 1:** The mix ratios of APC and anhydrite.

Specimen No.	APC Clinker (wt.%)	Anhydrite (wt.%)
G0	100	0
G1	85	15
G2	80	20
G3	75	25
